# Supplementary material for: Association of Cerebral Venous Thrombosis with mRNA COVID-19 Vaccines: A Disproportionality Analysis of the World Health Organization Pharmacovigilance Database
Source: Vaccines (Basel). 2022 May 18;10(5):799. doi: 10.3390/vaccines10050799 (PMC9145068; doi:10.3390/vaccines10050799)
Supplement: Supplementary file 1 [file vaccines-10-00799-s001.zip › vaccines-1681648-supplementary.pdf]

Supplementary Material

1. Supplementary Table S1. Mortality of patients with cerebral venous thrombosis after COVID-19 vaccination among vaccine types.

	BNT162b2 (n = 620)	mRNA-1273 (n = 136)	ChAdOx1 nCoV-19 (n = 757)	BNT162b2 vs. ChAdOx1 nCoV-19		mRNA-1273 vs. ChAdOx1 nCoV-19		BNT162b2 vs. mRNA-1273		mRNA-based vs. ChAdOx1 nCoV-19	
	Number (%)	Number (%)	Number (%)	Odds ratio (95% CI)	*P* value	Odds ratio (95% CI)	*P* value	Odds ratio (95% CI)	*P* value	Odds ratio (95% CI)	*P* value
Mortality	44 (7.0%)	5 (4.0%)	135 (18.0%)	0.35 (0.25–0.50)	< 0.001	0.18 (0.07–0.44)	< 0.001	2.00 (0.77–6.59)	0.60	0.32 (0.22–0.45)	< 0.001

Abbreviation: CI, confidence interval

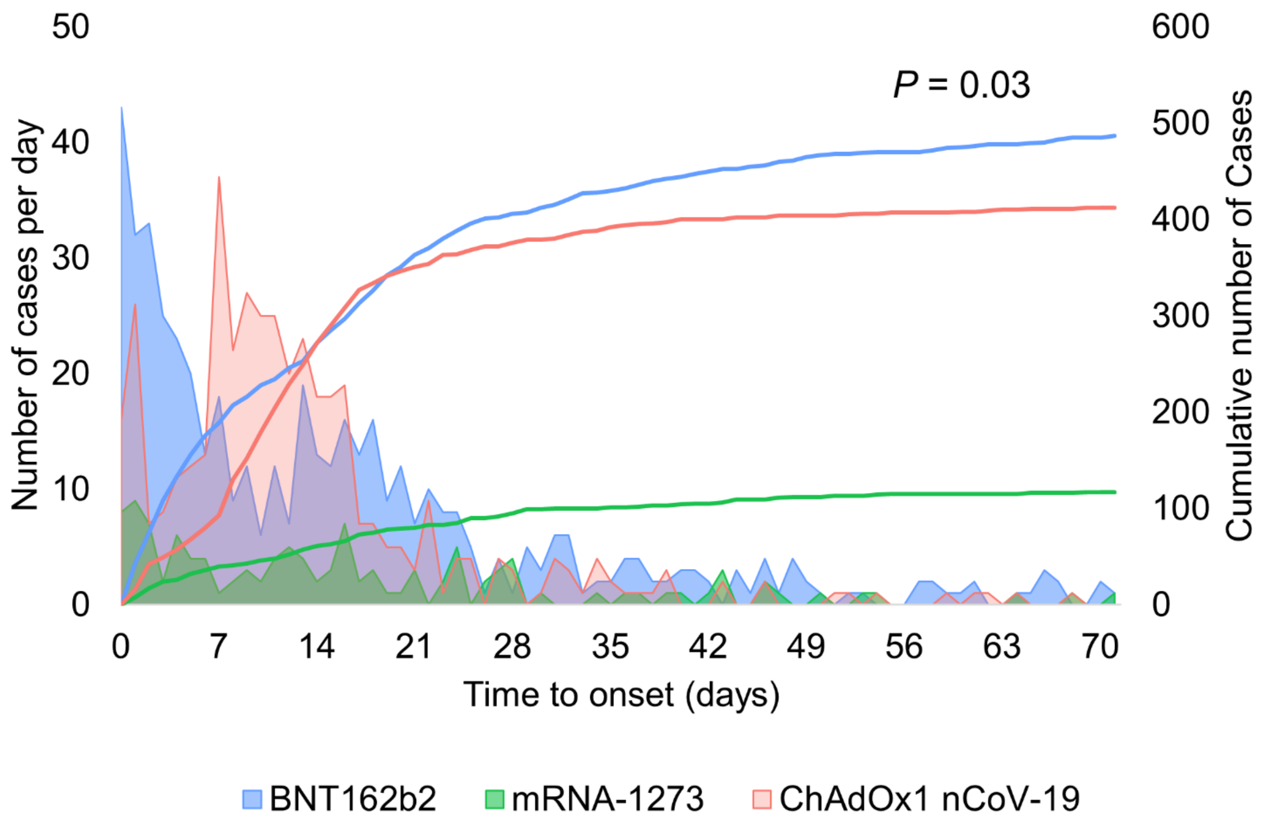

Supplementary Figure S1. The daily numbers of cerebral venous thrombosis cases and the cumulative frequency for all the vaccines for the entire period.

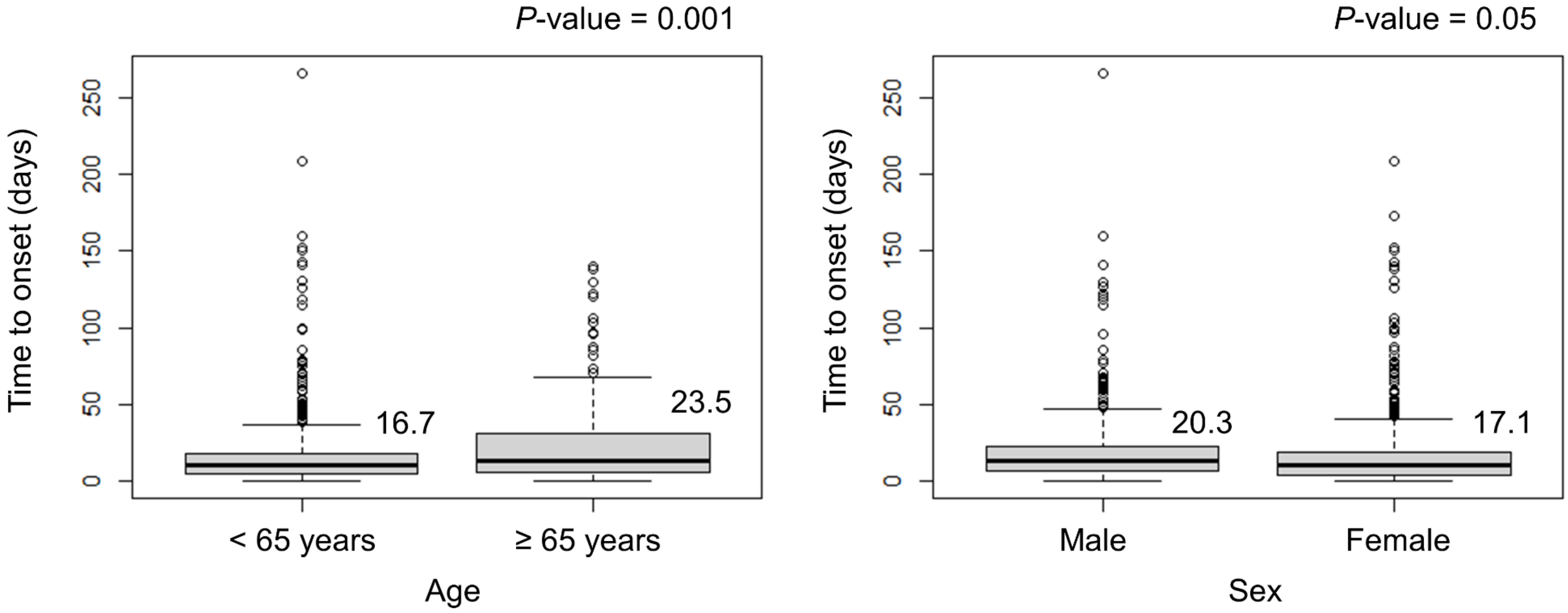

Supplementary Figure S2. The time to onset of cerebral venous thrombosis after COVID-19 vaccination between age and sex.
